# Serum protein coating enhances the antisepsis efficacy of silver nanoparticles against multidrug-resistant *Escherichia coli* infections in mice

**DOI:** 10.3389/fmicb.2023.1153147

**Published:** 2023-05-24

**Authors:** Huamao Du, Xiaoling Wang, Hongying Zhang, Heming Chen, Xiaoyu Deng, Yujing He, Huaze Tang, Fuchang Deng, Zhihong Ren

**Affiliations:** ^1^College of Biotechnology, Southwest University, Chongqing, China; ^2^Clinical Laboratory, Shanxi Academy of Traditional Chinese Medicine, Shanxi Traditional Chinese Medicine Hospital, Taiyuan, China; ^3^Chinese Center for Disease Control and Prevention, National Institute for Communicable Diseases Control and Prevention, Beijing, China

**Keywords:** protein corona, silver nanoparticles, multidrug-resistant bacteria, sepsis, pro-inflammatory cytokine

## Abstract

Antimicrobial resistance poses a significant threat to public health and social development worldwide. This study aimed to investigate the effectiveness of silver nanoparticles (AgNPs) in treating multidrug-resistant bacterial infections. Eco-friendly spherical AgNPs were synthesized using rutin at room temperature. The biocompatibility of both polyvinyl pyrrolidone (PVP) and mouse serum (MS)-stabilized AgNPs was evaluated at 20 μg/mL and showed a similar distribution in mice. However, only MS-AgNPs significantly protected mice from sepsis caused by the multidrug-resistant *Escherichia coli (E. coli)* CQ10 strain (*p* = 0.039). The data revealed that MS-AgNPs facilitated the elimination of *Escherichia coli (E. coli)* in the blood and the spleen, and the mice experienced only a mild inflammatory response, as interleukin-6, tumor necrosis factor-α, chemokine KC, and C-reactive protein levels were significantly lower than those in the control group. The results suggest that the plasma protein corona strengthens the antibacterial effect of AgNPs *in vivo* and may be a potential strategy for combating antimicrobial resistance.

## 1. Introduction

The increasing threat of drug-resistant bacteria poses a significant risk to human and animal health. The World Health Organization (WHO) has reported that the global drug-resistant microbial infection rate has reached 79%, which could lead to a severe condition of fatal sepsis in the absence of effective antibiotics (SEARO WHO South-East Asia Region, 2014). Therefore, finding novel antibiotics and antibacterial materials is of utmost importance. In this regard, silver nanoparticles (AgNPs) are a promising candidate due to their ability to induce the production of reactive oxygen species (ROS) and directly inactivate sulfhydryl respiratory enzymes (Rai et al., 2009; Joshi et al., 2020; Nene et al., 2021). It has been reported that AgNPs are highly efficient in killing hundreds of multidrug-resistant (MDR) bacteria *in vitro*, such as *Staphylococcus aureus, Pseudomonas aeruginosa*, and *Klebsiella pneumonia* (Chen et al., 2017; Kumar et al., 2019; Farouk et al., 2020; Slavin et al., 2021). While the cytotoxicity of AgNPs to mammalian cells led to their removal from over-the-counter use by the FDA in 1992, more recent *in vivo* toxicity tests have suggested that they may be a low-toxicity nanomaterial (Foldbjerg et al., 2015; Kennedy et al., 2018; Singh et al., 2018; Gan et al., 2020; Sofranko et al., 2021). To evaluate the potential clinical application of AgNPs in the treatment of MDR bacterial infections, we characterized the biosafety of AgNPs coated with mouse serum protein (MS-AgNPs) and used MS-AgNPs to treat mice infected with *E. coli* CQ10 strains that are resistant to 14 antibiotics, including cephalosporin and colistin (Bai et al., 2016). Our data suggest that it is possible to achieve a balance between biosafety and antibacterial effects *in vivo* using the serum protein coating method. This study provides preclinical data for the medical application of silver nanoparticles.

## 2. Materials and methods

### 2.1. Material

Dr. Xiong Yanwen donated the *Escherichia coli* (*E. coli*) CQ10 strain. Enzyme-linked immunosorbent assay (ELISA) kits for measuring mouse interleukin-6 (IL-6), tumor necrosis factor-α(TNF-α), chemokine KC, and C-reactive protein (CRP) were purchased from Wuhan Boshi Biological Company, China. Rutin, silver nitrate (AgNO_3_), and polyvinylpyrrolidone K3 (PVP) were purchased from Chongqing Chemical Reagent Company, China.

### 2.2. Experimental animal and ethics statement

The SPF-level Kunming mice (5 weeks old, male) purchased from the Animal Experimental Center of Chongqing Army Medical University were raised in a biosafety isolator. This study was approved by the Ethics Review Committee of Southwest University (permission number IACUC-20230228-17).

### 2.3. Preparation of AgNPs

AgNPs were synthesized in an eco-friendly manner by adding 1 mg/mL AgNO_3_ into a 0.1 mg/mL rutin solution (pH 10) and allowing the reaction to proceed in the dark at room temperature for 10 h. AgNPs were collected by centrifugation (12,000 × rpm for 20 min) and washed twice with deionized water, and then, large particles were removed by centrifugation at 5,000 × rpm for 30 min. We then adjusted the *OD*_404_ value of the silver colloid to 0.2 with a NanoDrop-2000 spectrophotometer (Thermo Fisher Scientific, USA). PVP-AgNPs were prepared by adding 50 μmol/L PVP solution, while MS-AgNPs were prepared by adding 1% mouse serum. Both types were washed three times with deionized water. The morphology of the AgNPs was visualized under a transmission electron microscope (TEM) at 20 kV, while the size distribution and Zeta potential were measured using a Zetasizer Nano ZS (Malvern, the UK). Finally, the component of the protein corona of MS-AgNPs was analyzed by mass spectrometry (Thermo Fischer Scientific, USA).

### 2.4. The antibacterial effect of AgNPs on the *E. coli* CQ10 strain *in vitro*

The minimal inhibitory concentration (MIC) and minimal bactericidal concentration (MBC) values of AgNPs were determined according to the CLSI M7-A7 and M27-A2 protocols, respectively. A range of AgNP concentrations (0, 2, 4, and 8 MIC) was added to a 1 × 10^6^ CFU/mL *E. coli* culture and incubated at 37°C. At 1, 3, 5, 7, 9, and 11 h, 200 μL cultures were mixed with 1 mmol/L Na_2_S. The live bacteria were counted on Luria-Bertani (LB) agar plates. This experiment was repeated three times, and the average values were used to generate time-kill curves.

### 2.5. Biocompatibility of silver nanoparticles

The hemocompatibility measurement was conducted in compliance with the GB/T16886.12-2005 ^[14]^ protocol. In summary, silver colloid concentrations of 10, 20, 40, and 80 μg/mL were blended with an equal volume of 5% mouse erythrocyte suspension. Deionized water and physical saline were used as controls. After incubating at 37°C for 30 min, the amount of released hemoglobin was quantified by the optical density at 540 nm (*OD*_540_) value. The experiment was repeated three times, and the hemocompatibility index was calculated using the formula: Hemolysis rate = [(*OD*_540_ sample–*OD*_540_ negative)/(*OD*_540_positive–*OD*_540_ negative)] × 100%.

Four sets of mice were administered with 0.2 mL of PVP-AgNPs, MS-AgNPs, and AgNO_3_ at a dose of 3 mg/kg body weight (b.w.) and 0.2 mL of PBS for the control group, with three mice per group. Blood was collected after 7 d, and the serum levels of aspartate transaminase (AST), alanine transaminase (ALT), blood urea nitrogen (BUN), and creatinine (CR) were measured. The liver, kidney, and spleen specimens were fixed with 4% paraformaldehyde, and the standard process of histologic section preparation was followed. The histologic examinations were performed using a Leica DM 6000B microscope (Wetzler, Germany).

### 2.6. Bio-distribution of AgNPs in mice

A total of 48 Kun-ming mice were randomly assigned to four groups. The experimental mice received a subcutaneous injection of AgNPs at a dose of 3 mg/kg b.w. in 0.2 mL, while the control mice were injected with an equal amount of AgNO_3_. The tissue distribution of silver was assessed on days 1, 3, 5, and 7 post-administration. The tissues and organs were accurately weighed and digested thoroughly in a 65% HNO_3_ solution at 100°C under a fuming cupboard. Subsequently, the dry matter was dissolved in 2 ml of a 2% HNO_3_ solution, and the silver content was determined using an atomic absorption spectrophotometer (Z5000, USA). The distribution of AgNPs was expressed as total Ag concentration, measured as μg/g weight.

### 2.7. Antibacterial effects of AgNPs in mice

#### 2.7.1. Animal experiment 1

A total of 25 mice were randomly divided into five groups, with one group serving as a healthy control. All test mice received an intraperitoneal injection of 1.5 median lethal doses (LD_50_) of CQ10 in 0.2 mL of phosphate buffer solution (PBS). After 1 h, the mice in the three test groups received a subcutaneous injection of 3 mg/kg b.w. of either PVP-AgNPs, MS-AgNPs, or AgNO_3_ in 0.2 mL, while the challenge control group received a 0.2 mL PBS injection. The morbidity and mortality of the mice were recorded for a period of seven days. The experiment was repeated three times under the same conditions, and the cumulative data were used to plot the survival curve using GraphPad Prism 5.

#### 2.7.2. Animal experiment 2

Due to the superior protective effect demonstrated by MS-AgNPs in the initial animal experiment, we sought to investigate their protection mechanism in terms of bacterial load and proinflammatory response. A total of 28 mice were randomly divided into two groups, both receiving an intraperitoneal injection of 1 LD50 CQ10 in 0.2 mL PBS. The experimental group received a subcutaneous injection of 3 mg/kg b.w. of AgNPs 1 h later, while the control group received 0.2 mL of PBS. The inflammatory response was evaluated at 4, 6, and 8 h after treatment. At each time point, three mice were randomly selected from each group, and tissue samples of the blood, liver, kidney, and spleen were collected and homogenized in PBS. Serially diluted solutions were then plated on LB agar plates (100 μL per plate) for colony counting, with 10 microliters of blood lysed in distilled water for enumeration. The concentrations of IL-6, TNF-α, CRP, and KC in serum were measured using an ELISA kit and following the manufacturer's instructions.

### 2.8. Statistical analysis of data

The mortality data of mice were analyzed using the log-rank (Mantel-Cox) test. All experimental data were presented as mean ± standard deviation (mean ± SEM). One-way analysis of variance (ANOVA) was used for intergroup comparisons. If *Levene's* test indicated homogeneity of variance, a least significant difference (LSD) test was used for pairwise comparisons. If the variance was not homogeneous, *Dunner's* T3 test was used instead. ^*^P < 0.05, ^**^P < 0.01.

## 3. Result

### 3.1. Characterization of silver nanoparticles

**Figure 1:**
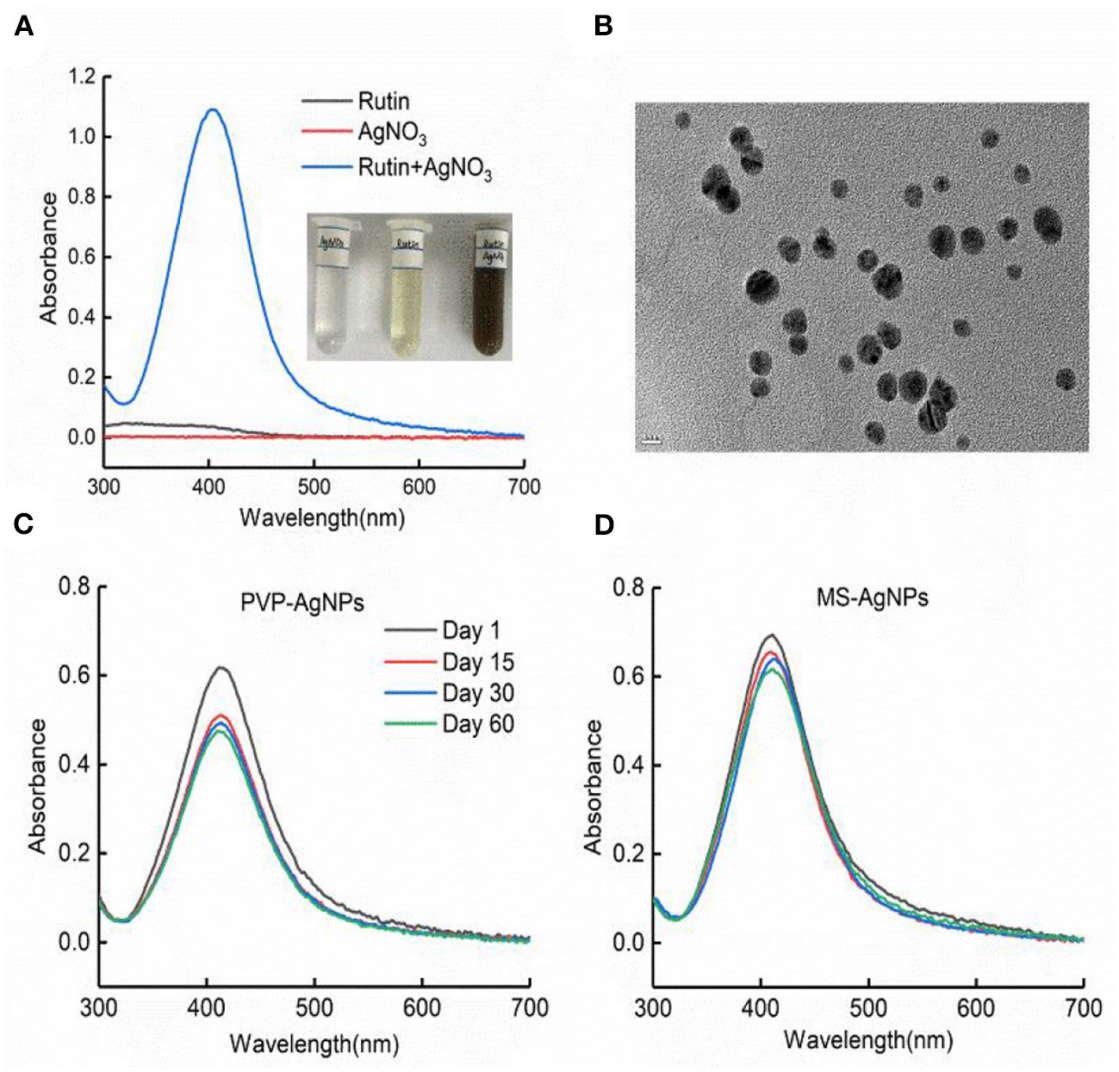
The characteristics of AgNPs synthesized with rutin. **(A)** Silver ion reduced by rutin to form nanoparticles; the plasmon resonance of AgNPs was shown by UV-vis absorbance at 401 nm with the NanoDrop 2000 spectrophotometer. **(B)** The morphology of AgNPs under TEM, bar = 10 nm. The stability of PVP-AgNPs **(C)** and MS-AgNPs **(D)** in 60 days at room temperature was detected with UV-vis absorbance.

**Figure 2:**
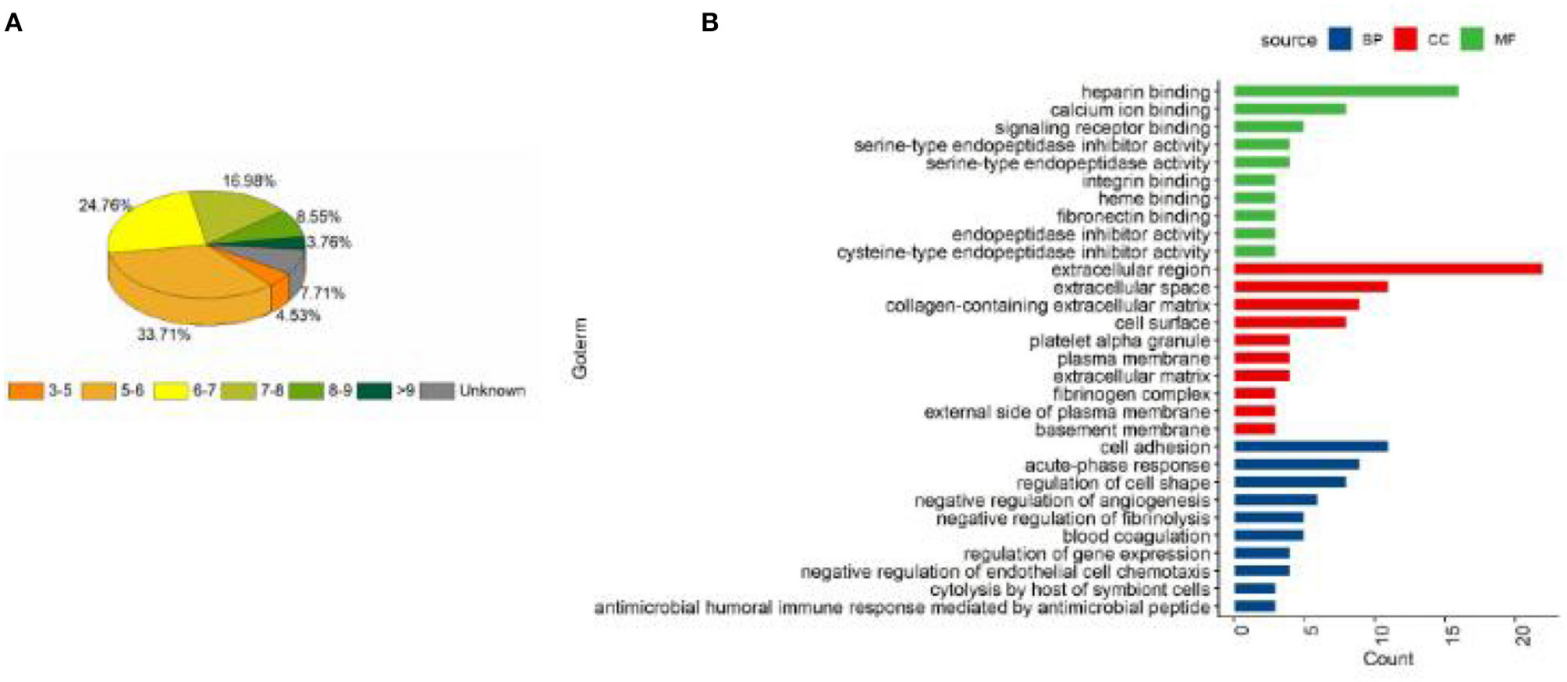
The distribution of pI and the biological function of the protein corona of MS-AgNPs. A total of 1% mouse serum was added to AgNPs and subjected to washing with ultrapure water, the protein components were determined with LC-MS. The distribution of pI **(A)** and biological function **(B)** of protein corona were analyzed.

AgNPs were synthesized in a rutin solution, which turned yellow and dark with an absorption peak at 404 nm (Figure 1A). The TEM visualization revealed that AgNPs had a spherical shape and a primary size of 2–20 nm (Figure 1B). Dynamic light scattering measurements showed that the hydrodynamic size was 72.7 ± 1.2 nm. When stabilized with PVP or protein corona, the hydrodynamic size increased to 124.8 ± 2.6 nm and 119.7 ± 0.9 nm, respectively. Electrophoresis light scattering data revealed a zeta potential value of −9 ± 1.2 mV and −12.9 ± 2.3 mV, respectively (Table 1). The UV-vis detection conducted over a 2-month period indicated that the AgNPs coated with protein corona were more stable compared to the PVP-AgNPs (Figures 1C, D). LC-MS analysis revealed the presence of approximately 125 serum proteins in the protein corona of MS-AgNPs, with complement components and albumin being the most abundant, each accounting for 10% (Table 2). GO analysis showed that these proteins were involved in all three types of metabolic pathways: biological process, cellular component, and molecular function. Heparin-binding, extracellular region, and cell adhesion were among the top three major functions. Since the formation of the protein corona is dependent on electrostatic interaction, we analyzed their isoelectric point. The results showed that the number of acidic proteins (pI 3–6) was relatively high, accounting for 38%, neutral proteins (pI 6–8), accounting for 40%, and that had a pI value above 8, accounting for only 12% (Figure 2). These data supported the negative zeta potential value obtained by ELS.

**Table 1:** Comparison of the size and Zeta potential of AgNPs.

**Sample**	**Hydro diameter (nm)**	**Zeta potential (mV)**	**PDI**
AgNPs	72.7 ± 1.2	−19.2 ± 0.5	0.49 ± 0.01
PVP-AgNPs	124.8 ± 2.6	−9.0 ± 1.2	0.53 ± 0.01
MS-AgNPs	119.7 ± 0.9	−12.9 ± 2.3	0.47 ± 0.01

**Table 2:** The major components of the protein corona of MS-AgNPs.

**Protein names**	**Ratio (%)**
Serum albumin	10.1
complement (C3, C4, H, B)	10.1
Gelsolin	4
Thrombospondin-1	3.7
Serotransferrin	2.6
Histidine-rich glycoprotein	2.5
Hemopexin	2.5
β-2-glycoprotein 1	2.2
α-2-macroglobulin	1.4
Plasminogen	1.3
Serine protease inhibitor A3K	1.3
CD5 antigen-like	1.3
Coagulation factor V	1.2
Fibronectin	1
Prothrombin	1

### 3.2. Time-dependent bactericidal effect of silver nanoparticles

**Figure 3:**
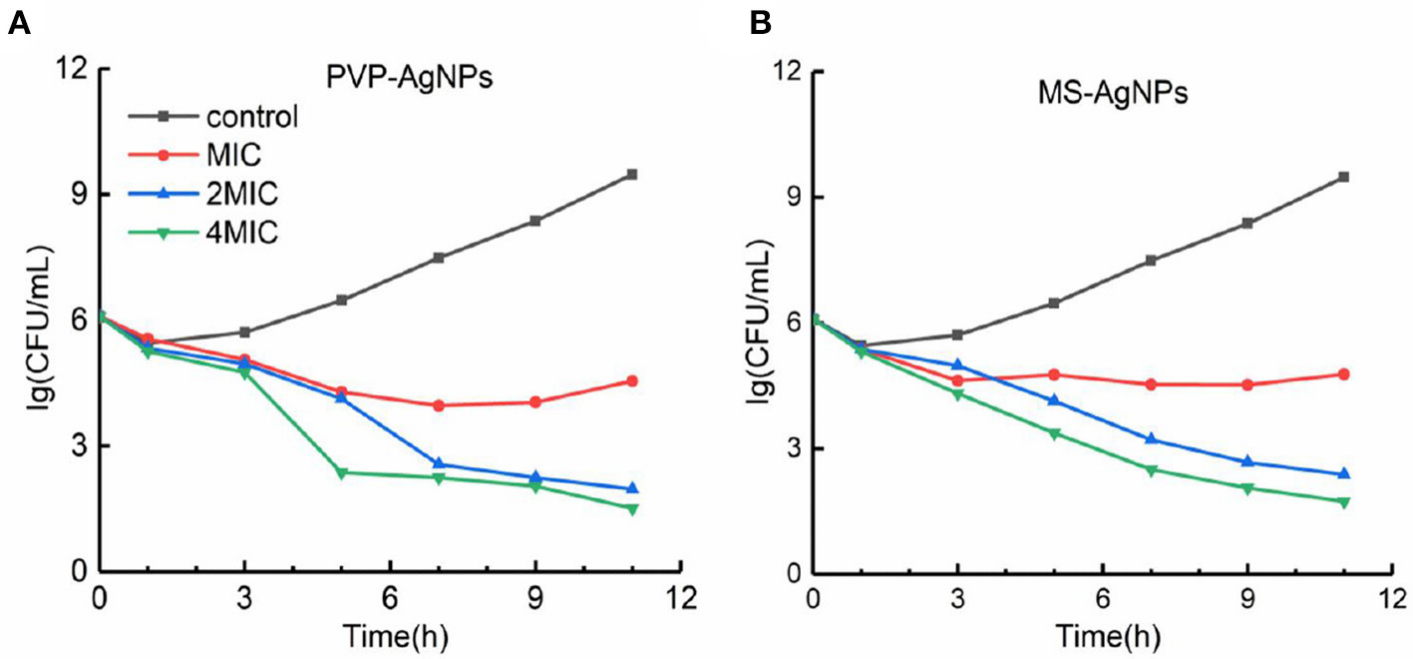
**(A, B)** The time-kill curve of AgNPs against the *E. coli* CQ10 strain. A suspension of 10^6^ CFU/mL *E. coli* CQ10, a multidrug-resistant clinical isolate, was treated with serial concentrations of 0, 1, 2, 4 MIC PVP-AgNPs, or MS-AgNPs, and the live *E. coli* in the samples at each time point was counted on LB agar plates after neutralizing with 1 mM Na_2_S.

With a determination of the antibacterial efficiency of PVP-AgNPs and MS-AgNPs against 10^6^ CFU/mL *E. coli* CQ10, our results showed that the MIC values for PVP-AgNPs and MS-AgNPs were 8 μg/mL and 16 μg/mL, respectively, while the MBC values were 16 μg/mL and 32 μg/mL, respectively. Both MBC/MIC ratios were equal to 2. We found no significant difference in the bactericidal efficiency of 2 MIC and 4 MIC of silver nanoparticles at each time point within 12 h. However, the bactericidal efficiency increased significantly with time extension, indicating that both types of AgNPs exhibited time-dependent antibacterial activity (Figure 3).

### 3.3. The biocompatibility of AgNPs

**Figure 4:**
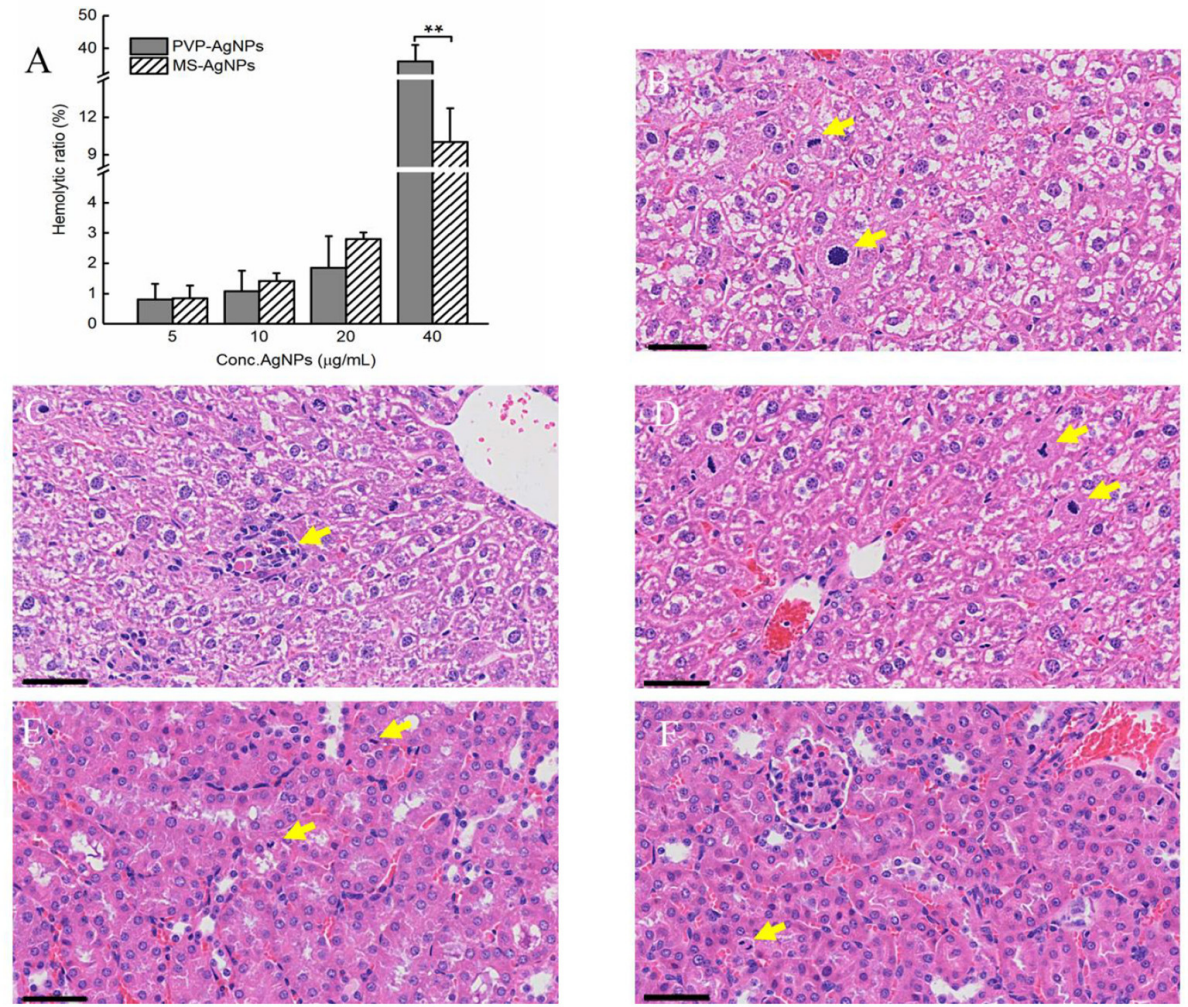
The hemocompatibility of AgNPs and histological examination of mice (*n* = 3). A serial concentration of AgNPs was mixed with 5% mouse erythrocyte suspension and the degree of erythrocyte lysis was determined using the *OD*_540_ of the supernatant. Each reaction had three replicates, and the experiment was repeated three times. The data were expressed as mean ± SD and analyzed by one-way ANOVA tests, *p* = 0.001 at concentration of 40 μg/mL. ^*^P < 0.05; ^**^P < 0.01. **(A)** The biocompatibility of AgNPs *in vivo* was assessed by histological examination of the livers and kidneys of mice. One dose of AgNPs, 3 mg/kg body weight(b.w.), was injected subcutaneously, and 7 days later, liver and kidney specimens were collected when mice were under anesthesia. Mild-level histological changes were observed in each group, such as hepatocyte nuclear division **(B, D)**, hepatocyte necrosis **(C)**, and renal tubular epithelial cell nuclear division **(E, F)**, without severe histological lesions. All tissue sections were stained with hematoxylin and eosin, bar = 50 μm.

The hemocompatibility of nanomedicine was assessed by its hemolytic effect. The hemolytic ratio of two types of AgNPs increased with higher concentration, both less than 5% at the concentration of 20 μg/mL, while PVP-AgNPs caused a significantly higher hemolytic ratio than MS-AgNPs at 40 μg/mL (*p* = 0.001, Figure 4A). The *in vivo* biosafety of AgNPs was evaluated by administering a dose of 3 mg/Kg b.w. to mice over a period of 7 d and assessing for acute toxicity. Serum levels of ALT, AST, urea nitrogen, and creatinine in experimental mice increased slightly compared with controls by 7 d, but without statistical significance (Table 3). The histological examination results revealed the presence of nuclear division, hepatocyte necrosis, and inflammatory cell infiltration in both the test groups and the control, indicating slight or mild-level histological changes without severe histological lesions (Table 3). These histological changes appear to be unrelated to the dose of silver administered in the study since they are common spontaneous diseases in mice (Figures 4B–F).

**Table 3:** Histological examinations and serum makers of the liver and the kidney (*n* = 3).

**Groups**	**Liver**				**Kidney**		
	**HC nuclear division**	**HC necrosis**	**ALT (U/L)**	**AST (U/L)**	**REC nuclear division**	**UN (mg/dl)**	**CR (**μ**mol/L)**
Control	1 (1/3), 2 (1/3)	1 (2/3)	37.4 ± 5.1	97.0 ± 0.7	0 (3/3)	12.1 ± 0.7	18.3 ± 0.5
PVP-AgNPs	2 (3/3)	1 (1/3)	41.1 ± 10.8	94.7 ± 12.4	1 (2/3)	13.4 ± 1.8	22.7 ± 2.6
MS-AgNPs	2 (3/3)	1 (2/3)	40.6 ± 10.0	143.2 ± 41.6	1 (1/3)	14.3 ± 2.9	24.5 ± 5.1
AgNO_3_	1 (1/3), 2 (2/3)	1 (1/3)	35.6 ± 4.9	87.1 ± 11.9	0 (3/3)	13.8 ± 0.9	21.7 ± 0.5
*P* value	-	-	0.891	0.192	-	0.649	0.251

Histological standard: 0, normal; 1, slight; 2, mild; 3, moderate; and 4, severe. HC, hepatocytes; REC, renal tubular epithelial cells; ALT, alanine transaminase; AST, aspartate transaminase; UN, urea nitrogen; CR, creatinine.

### 3.4. Distribution of AgNPs *in vivo*

**Figure 5:**
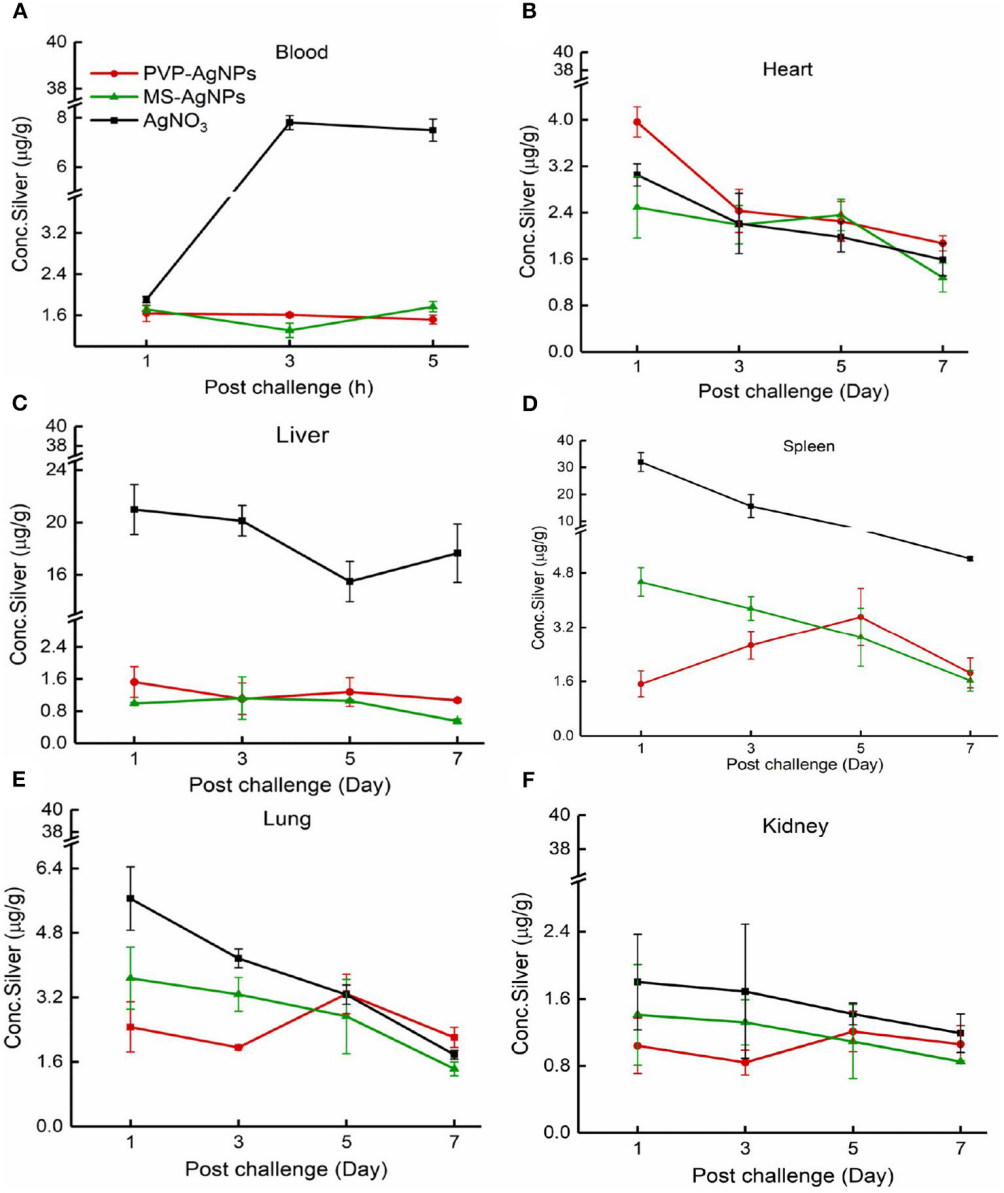
**(A–F)** The distribution of silver in mouse tissue. A single dose of AgNPs or AgNO_3_ was injected at 3 mg/Kg b.w., the total silver concentration in the heart, liver, spleen, lung, and kidney was measured 1, 3, 5, and 7 days later, except that the serum concentration was tested at 1, 3, and 5 h. The tissue samples were collected from three mice at each time point in one group. The data were expressed as mean ± SD.

We conducted a study to determine the absorption and accumulation of PVP-AgNPs and MS-AgNPs in blood and various organs. Our results showed that both types of AgNPs were quickly absorbed into the blood and maintained a concentration of 1.62 ± 1.21 μg/g within 5 h. In the MS-AgNPs group, the concentration of silver in the spleen, liver, kidney, and heart decreased continuously over 7 d, with the largest decrease observed in the spleen and the smallest in the liver. In contrast, in the PVP-AgNPs group, the accumulation of silver in the spleen, kidney, and lung reached its highest level on the 5th day, with concentrations ranging between 0.55 and 2.21 μg/g on the 7th day. Notably, in the PVP-AgNPs group, the administration of AgNO_3_ solution resulted in higher concentrations of silver in the blood, spleen, and liver, which were maintained for a longer period compared to both AgNP groups (Figure 5).

### 3.5. Therapeutic effect of a single dose of AgNPs

**Figure 6:**
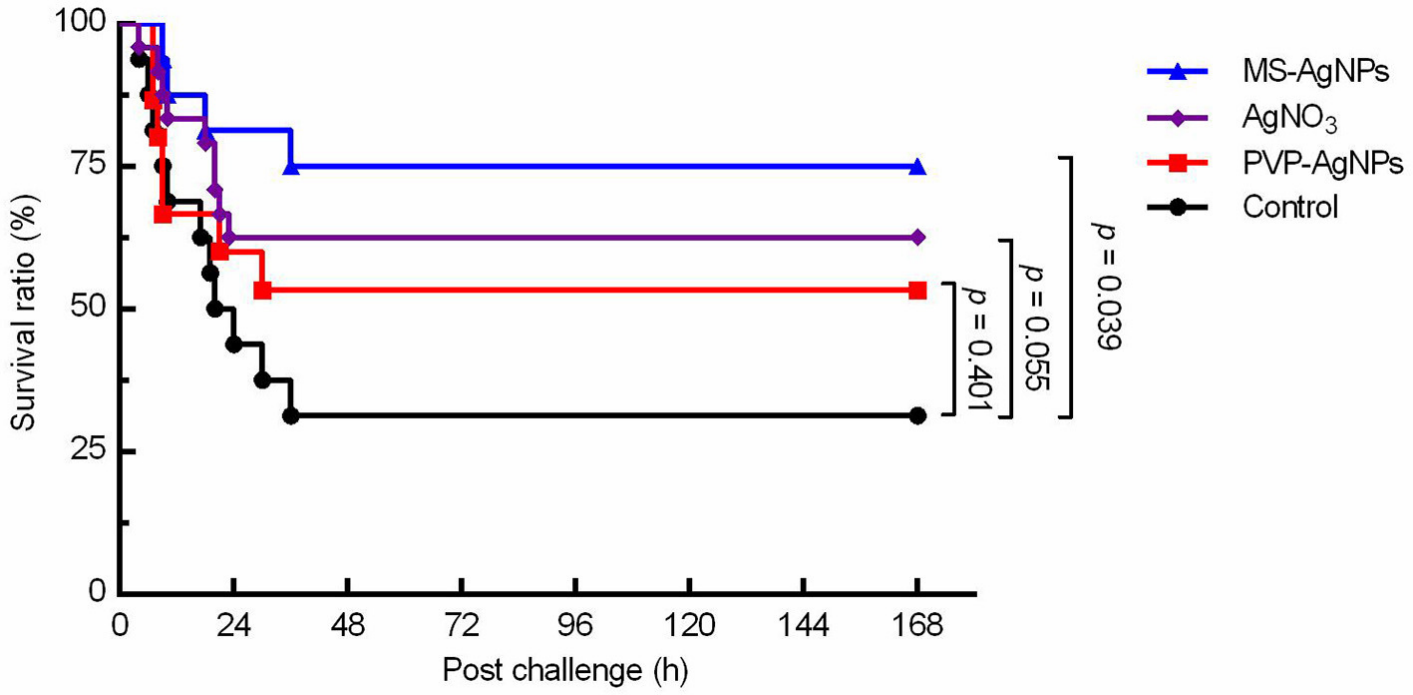
The survival curve of mice challenged with the MDR *E. coli* CQ10 strain. All mice were challenged with 1.5 LD_50_ CQ10, and 1 h later, 3 mg AgNPs/kg b.w. were injected subcutaneously. PBS and the same mass of AgNO_3_ were taken as controls. The experiments were repeated three times, and the data were analyzed by the log-rank (mantel-Cox) test.

The LD_50_ value of the *E. coli* CQ10 strain for Kunming mice was determined to be 3.12 × 10^8^ CFU through intraperitoneal challenges. Following the challenge, mice exhibited shivering, vertical hair, and poor coordination. The control group had a mortality rate of 70%, which was significantly higher than the MS-AgNPs-treated group, which had a mortality rate of 25% (*p* = 0.039). The therapeutic effect of AgNO_3_ was slightly lower than that of MS-AgNPs, with a mortality rate of 37.5% (*p* = 0.058), and PVP-AgNPs group reached to 46.7% (*p* = 0.401), being the worst outcome. It is important to note that all mice in each group died within 48 h, indicating sepsis (Figure 6).

**Figure 7:**
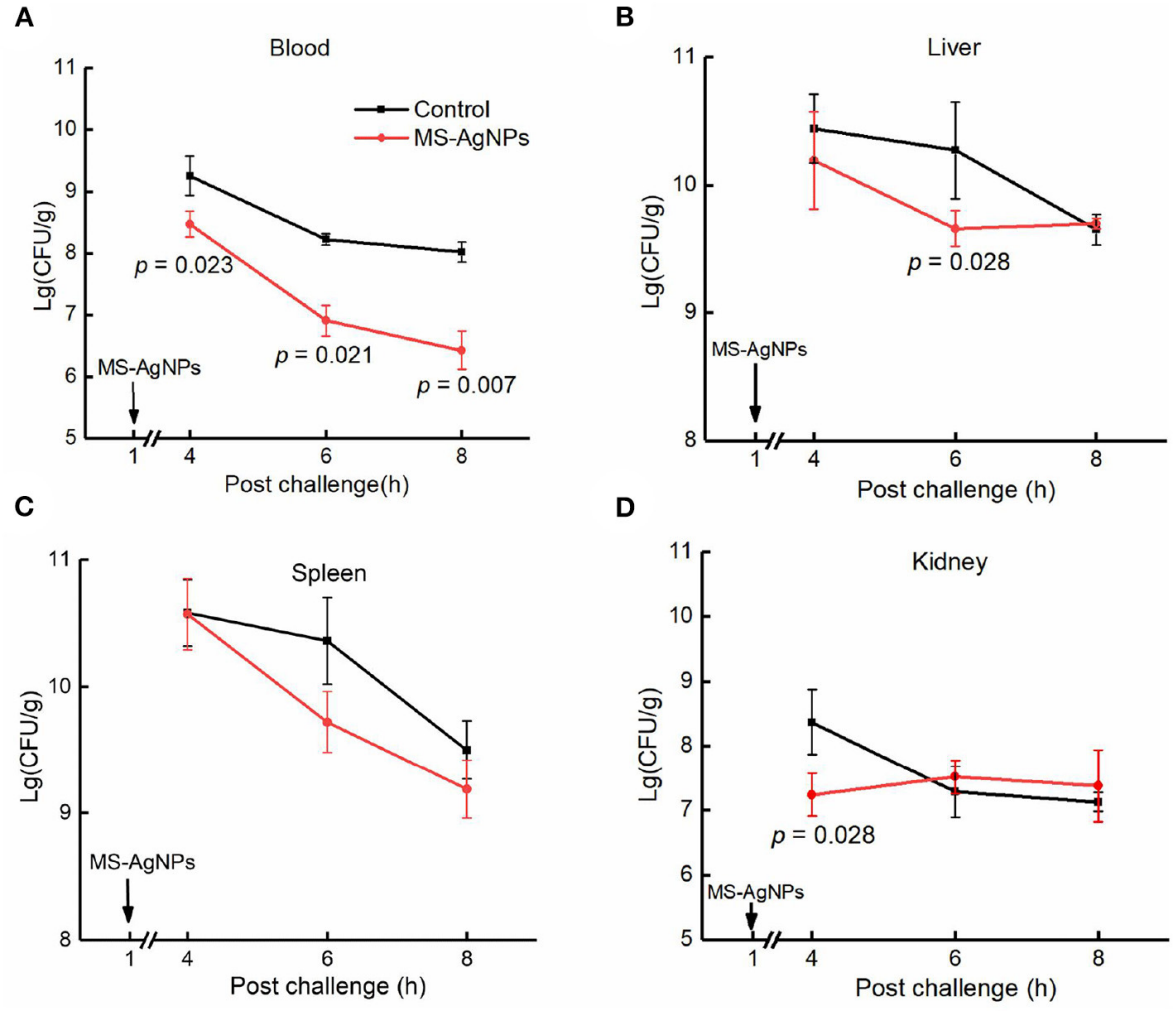
**(A–D)** The bacterial burden in tissues after challenge. All mice were injected i.p. 3.1 × 10^8^ CFU *E. coli* CQ10 strain; 1 h later, 3 mg MS-AgNPs per kg b.w. were injected in the test group and PBS for control. At time points of 4, 6, and 8 h, three mice were anesthetized, and the tissue samples were precisely weighted, respectively. The tissues were homogenized and diluted with PBS. A total of 100 μl of suspension were plated on LB agar plates; each dilution was set on three plates. The data were expressed as mean ± SD and analyzed by one-way ANOVA followed by LSD.

To investigate the mechanism by which MS-AgNPs improve the survival rate of mice, we conducted tests to measure bacterial burden and major proinflammatory cytokines during the early stage of infection. Our findings revealed a significant decrease in bacterial burden across all tested organs during the first 4 to 8 h after infection. Specifically, the bacterial blood burden was significantly lower in the test group compared to the control (*p* = 0.007), with a 100-fold decrease in CQ10 in the former and a 10-fold decrease in the latter. The spleen and liver showed the same level of bacterial burden in the 4th h after the challenge (about 10^10.2 − 10.5^CFU/g). In the 8th h, the concentrations of CQ10 decreased by 10 and 5%, respectively. While MS-AgNPs enhanced the reduction of *E. coli* in the spleen and liver, there was no significant effect observed in the kidney (Figure 7).

**Figure 8:**
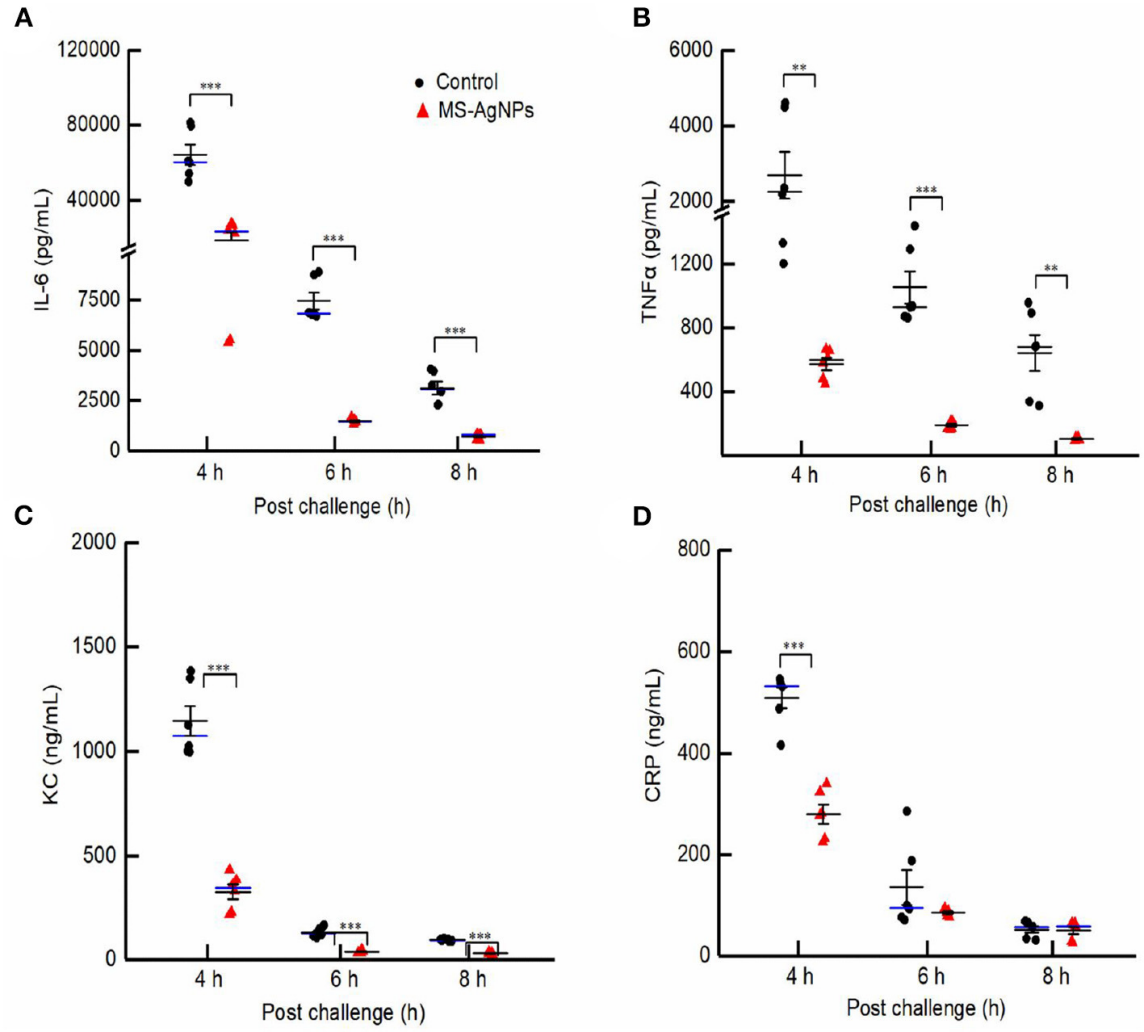
**(A–D)** The dynamic of proinflammatory cytokines in the early-stage post challenge. All mice were injected i.e., 3.1 × 10^8^ CFU *E. coli* CQ10 strain; 1 h later, 3 mg MS-AgNPs per kg b.w. were injected in the test group and PBS for control. At 4, 6, and 8 time points, three mice had anesthesia and blood, respectively. The levels of IL-6, TNF-α, KC, and CRP were measured with sandwich ELISA. The data were expressed as mean ± SD and analyzed by one-way ANOVA followed by LSD, ^**^P < 0.01; and ^***^P < 0.001.

In this study, despite administering a single dose of MS-AgNPs 1 h after the challenge, a significant degree of inflammation relief was observed (Figure 8). In the 5th h after the challenge, the test group exhibited blood levels of IL-6, TNF-α, KC, and CRP of 23.2 ng/mL, 0.6 ng/mL, 343.5 ng/mL, and 280.2 ng/mL, respectively. These levels decreased by 27.6, 5.1, 10.1, and 3.9 times in the 8th h, respectively. In contrast, the infected control group had 60.2 ng/mL, 2.3 ng/mL, 1,073.9 ng/mL, and 532.2 ng/mL levels of IL-6, TNF-α, KC, and CRP, respectively, in the 5th h after infection, and these levels decreased by 18.5, 2.3, 10.5, and 8.4 times at the 8th h, respectively. The levels of inflammatory-related cytokines showed significant differences at each time point (*P* < 0.01), except for the levels of CRP at 6 and 8 h after the challenge. The decreased inflammatory response and bacterial load in the MS-AgNPs group supported the high survival rate of mice.

## 4. Discussion

*Escherichia coli* was previously believed to be typically transmitted through food, but it has since been found to cause a wide range of infections beyond the intestinal tract, including urinary tract, wound, and systemic infections (Chiurchiu et al., 2003; Mokady et al., 2005; Lienemann et al., 2012; Buvens et al., 2013; Bai et al., 2016; Jenssen et al., 2016; Dennhardt et al., 2018; Kato et al., 2019; Habets et al., 2021). Certain strains, such as O2 and O78, can even lead to meningitis and septicemia. The WHO has identified carbapenem-resistant and extended-spectrum β-lactamase (ESBL)-producing *Enterobacteriaceae* as top priority pathogens in 2017. Our study focused on the Shiga-like toxin-producing *E. coli* CQ10 strain, specifically the O2:H32 serotype, which has shown resistance to cephalosporin, colistin, and 12 other antibiotics (Bai et al., 2016). This clinical isolate is particularly concerning due to its high risk.

Sepsis caused by bacterial infection is a leading cause of death in hospitalized patients. One of the most significant biomarkers of sepsis is the proinflammatory cytokines IL-6 and TNF-α, which are closely related to patient outcomes. Viral infections can also induce a severe inflammatory response, leading to acute death, as observed in the hepatitis virus experimental infection and the COVID-19 pandemic outbreak (Dong Kim et al., 2007; Chen et al., 2020). High serum levels of IL-6 and TNF-α can cause strong immunopathological reactions and tissue damage and increase the likelihood of death from shock, especially in experimental animals (By Hack et al., 1989; Jin et al., 2001; Ye et al., 2009; Goodman and Brett, 2017). Therefore, the Application Guidelines for Saving Sepsis Patients recommend controlling infection levels as quickly as possible (Gnanadhas et al., 2013).

In this study, MS-AgNPs were subcutaneously injected, resulting in a sharp decline in both the bacterial burden and serum inflammatory cytokines within 8th h. The bacterial burden in the blood and spleen decreased by 100-fold between the 8th and 4th h after the challenge with 1.5 LD_50_ CQ10 (Figures 7A, C). The production of IL-6, TNF-α, CRP, and chemokine KC in the MS-AgNPs-treated group were significantly lower than those of the control group at three time points (Figure 8). These results were consistent with the previous report (Gnanadhas et al., 2013). These results demonstrate that the antibacterial effect of MS-AgNPs can avoid over-activating the inflammatory response in mouse models, allowing them to survive cytokine storms.

Nanomaterials interact with amphoteric proteins in biological fluids, forming a protein corona due to their charge (Durán et al., 2015). This corona is a dynamic process, with tightly bound proteins in the inner layer and loosely and reversibly bound proteins in the outer layer (Miclăuş et al., 2016). The formation of a protein corona can affect the original characteristics of nanomaterials, such as the loss of targeting ability in some molecules (Salvati et al., 2013). However, albumin coating can improve silica nanoparticles' water solubility and stability, reduce non-specific degradation, and prolong their circulation *in vivo* (Mariam et al., 2016).

The composition of the protein corona can also affect the antibacterial efficiency, biological distribution, and half-life of AgNPs, reducing their uptake by mammalian cells and, thus, decreasing their cytotoxicity. For example, human cells uptake fewer AgNPs with a human serum protein corona than murine cells (Shannahan et al., 2014; Brown et al., 2015; Kennedy et al., 2018). In this study, the therapeutic effects of PVP and MS-stabilized AgNPs were compared, and it was found that MS-AgNPs had higher antibacterial efficiency *in vivo* than PVP-AgNPs, significantly improving the survival rate of infected mice (Figure 6). Although the MIC value of MS-AgNPs was higher than that of PVP-AgNPs, the protein corona of MS-AgNPs improved its antibacterial impact by involving complement and immunoglobulin in immune defense. However, the antibacterial mechanism *in vivo* remains to be further detailed.

The cytotoxicity and genetic toxicity of AgNPs have been extensively researched, and their main effects include cell membrane damage, cell cycle arrest, DNA damage, apoptosis, cytoskeleton damage, and autophagy (AshaRani et al., 2009; Fageria et al., 2017; Barbalinardo et al., 2018). The toxicities *in vivo* have also been assessed experimentally in mice, and the results were diverse due to no standard protocol (Skalska and Struzynska, 2015). A 28-day oral exposure to 9 mg/kg b.w. caused an increase in dopamine and 5-hydroxytryptamine in the brain (Hadrup et al., 2012) while a dose of 1 mg/kg b.w. per day did not result in significant toxicity in histological examination or blood markers (Qin et al., 2017). Moreover, 14-week repeated intranasal exposure of 0.1 mg/Kg b.w. caused significant body weight loss with neuroglial cell activation (Yin et al., 2015). A single exposure of 50 mg/Kg b.w. caused brain edema, glial cell activation, and loss of myelinated fibers (Sharma et al., 2009). The toxicity of AgNPs *in vivo* depends on the quantity of uptake by mice. In this study, at a single dose of 3 mg/kg b.w., the silver content in the spleen, liver, kidney, and lung of mice decreased by 7 d, and no severe histological lesion was observed in the liver and the kidney (Figure 4). The levels of ALT, AST, urea nitrogen, and creatinine in the blood increased slightly (Table 3). Most importantly, the severe infection of the MDR CQ10 strain could be controlled with a single dose of 3 mg/Kg b.w. Given the old saying, “When faced with two evils, choose the lesser one,” AgNPs could be one of the most valuable candidates to treat fatal infections caused by MDR or pan-drug-resistant bacteria in wild animals, pets, and specific human cases.

## Data availability statement

The raw data supporting the conclusions of this article will be made available by the authors, without undue reservation.

## Ethics statement

The animal study was reviewed and approved by the Ethics Review Committee of the National Institute for Communicable Disease Control and Prevention at the Chinese Center for Disease Control and Prevention (Beijing, China).

## Author contributions

Conception and design and provision of study materials: HD. Administrative support: HD, ZR, and XW. Investigation and collection and assembly of data: HZ, HC, and HT. Data analysis and interpretation and visualization: XD, YH, FD, and HT. All authors writing the manuscript and provide final approval.
